# Diagnosing Extrapulmonary Sarcoidosis and the Implications of Diagnosis on Military Service

**DOI:** 10.7759/cureus.47115

**Published:** 2023-10-16

**Authors:** Caroline E Moore, Landry Marshall

**Affiliations:** 1 Internal Medicine, Naval Medical Center San Diego, San Diego, USA; 2 Internal Medicine, Naval Medical Center Camp Lejeune, Camp Lejeune, USA

**Keywords:** pancytopenia, diagnostic delay, non-caseating granuloma, clinical reasoning, military medicine, active-duty military personnel, extrapulmonary sarcoid, renal sarcoidosis

## Abstract

Sarcoidosis is a systemic inflammatory disease that can cause granulomatous infiltration of almost all organs and tissues which allows for a wide variety of presentations that may overlap with other disease processes. Renal sarcoidosis is a much rarer site of extrapulmonary involvement and may present as hypercalcemia, tubular or glomerular dysfunction, and/or granulomatous interstitial nephritis. Prompt diagnosis of sarcoidosis is crucial for initiating appropriate treatment and avoiding organ dysfunction.

Herein, we describe a case of an armed forces service member who developed extrapulmonary sarcoidosis and renal sarcoidosis with acute complications refractory to glucocorticoids requiring adalimumab. The case highlights and emphasizes a rare manifestation of extrapulmonary sarcoidosis, the importance of avoiding premature closure of the differential diagnosis to avoid diagnostic delay and treatment imitation, and the unique clinical reasoning that occurs in active-duty personnel where diagnoses and subsequent treatments can have career implications and affect the ability of the service member to maintain the ability to deploy worldwide.

## Introduction

Sarcoidosis is a chronic, systemic inflammatory disease of unknown etiology characterized by the abnormal development of noncaseating granulomas within organs and tissues [1]. African Americans are disproportionately affected by this disease compared to Caucasian individuals, with an estimated incidence of ~36 per 100,000 [2]. Additionally, while most patients experience remission of their disease, African-American patients are also much more likely to develop a chronic, severe disease course [2,3]. The diagnosis of sarcoidosis can be made with supportive clinical, laboratory, and radiographic data with or without a tissue biopsy, and exclusion of other causes of granulomatous diseases such as mycobacterial infections is imperative [2]. The diagnosis can be delayed as the presentation of this disease can be heterogeneous and non-specific [4].

Any organ can be affected in sarcoidosis, and approximately 30%-50% of patients have extrapulmonary involvement [1]. The pulmonary system is the most common site of involvement whereas renal involvement is much rarer [1]. Less than 1% of individuals present with renal sarcoidosis and approximately 1% to 5% of individuals with sarcoidosis have their kidneys affected [1]. The most common subtype of renal sarcoidosis is granulomatous interstitial nephritis which most often occurs in the context of systemic disease [2]. However, hypercalcemia, proteinuria, microscopic hematuria, and hypergammaglobinemia can all be associated findings [2]. The mainstay of treatment for extrapulmonary sarcoidosis is high-dose glucocorticoids and if ineffective, patients may require immunosuppressive therapy such as tumor necrosis factor-alpha inhibitors (TNF-alpha) [1,3].

Importantly, when systemic diseases such as sarcoidosis develop in an active-duty service member, it may have implications on the individual’s future military career and worldwide deployability which is essential to maintaining a medically ready and able force. Specific diagnoses and anticipated treatment courses may inhibit a service member from having full operational capabilities and maintaining military-based competencies, and thus, require medical separation from service. The case presented herein highlights the heterogenetic presentation of sarcoidosis as well as stresses the importance of avoiding premature closure of the differential diagnosis to avoid diagnostic delay. This case also gives insight into military medicine decision-making, as it is critical for meeting the overall military’s mission that service members are fully medically ready and capable of executing orders around the globe.

## Case presentation

A previously healthy 29-year-old African-American active-duty male presented to his primary care physician with the chief complaint of concern for high blood pressure as previous outpatient readings had been elevated. He denied experiencing headaches, visual changes, shortness of breath, chest pain, or lower extremity swelling and described eating a well-balanced nutritional diet and regularly participating in exercise. He reported an intentional weight loss of five pounds over the last six months and denied fatigue or decreased endurance. His past medical history was significant for sickle cell trait and right wrist pain secondary to a right scaphoid wrist nonunion fracture that occurred six months prior without an inciting event or trauma that was associated with significant intramedullary cystic changes, a large radiocarpal joint effusion, and loss of bony architecture on radiographs and magnetic resonance imaging (MRI) with and without contrast. He had undergone an anterior and posterior interosseous neurectomy with distal pole excision for this issue and was engaged in occupational therapy. His family history was significant for hypertension in his mother and maternal grandmother but denied any other family history. He denied alcohol use but reported daily e-cigarette/vaping use with nicotine. On physical examination, his vitals were within normal limits besides his blood pressure, which was 153/101 mmHg. He was in no acute distress with a regular heart rate and regular rhythm and his lungs were clear to auscultation. The rest of his examination was unremarkable. His initial laboratory evaluations ordered by the outpatient provider are listed in Table 1.

**Table 1:** Initial Outpatient Laboratory Parameters cells/mm^3^: cells per cubic millimeters; g/dL: grams per deciliter; cells/µL: cells per microliter; mmol/L: millimoles per liter; mg/dL: milligrams per deciliter; U/L: units per liter

Parameter	Observed Value	Normal Range
White Blood Cells (WBC)	2100 cells	3200-10,800 cells/mm^3^
Segmented Neutrophils	62.8%	44.5-78.4%
Hemoglobin	7.7 g/dL	13.1-18.6 g/dL
Platelets	112,000 cells/µL	150,000-350,000 cells/µL
Serum Sodium	133 mmol/L	136-145 mmol/L
Serum Potassium	5.1 mmol/L	3.5-5.0 mmol/L
Serum Chloride	103 mmol/L	98-107 mmol/L
Serum HCO_3_^-^	28 mmol/L	21-32 mmol/L
Serum Urea	37 mg/dL	7-18 mg/dL
Serum Creatinine	4.1 mg/dL	0.7-1.3 mg/dL
Random Blood Glucose	148 mg/dL	70-100 mg/dL
Serum Calcium	12.3 mg/dL	8.5-10.1 mg/dL
Corrected Calcium	13.2 mg/dL	
Serum Total Protein	8.8 g/dL	6.4-8.2 g/dL
Serum Albumin	2.9 g/dL	3.4-5.0 g/dL
Serum Phosphorus	3.3 mg/dL	2.5-4.9 mg/dL
Serum Magnesium	2.2 mg/dL	1.6-2.6 mg/dL
Serum Total Bilirubin	0.7 mg/dL	0.2-1.0 mg/dL
Serum Alkaline Phosphatase	120 U/L	45-117 U/L
Serum Alanine Aminotransferase (ALT)	25 U/L	16-61 U/L
Serum Aspartate Aminotransferase (AST)	78 U/L	15-37 U/L
Uric Acid	9.6 mg/dL	3.5-7.2 mg/dL
Lactate Dehydrogenase (LDH)	658 U/L	87-241 U/L

**Figure 1:**
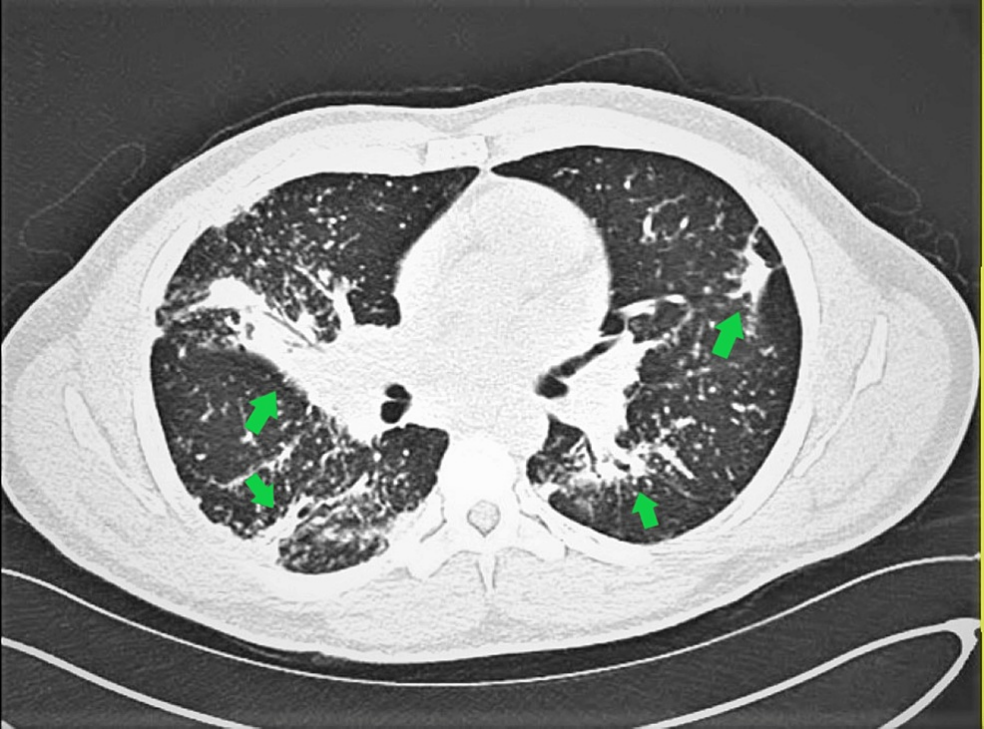
Pulmonary involvement of sarcoidosis Computed tomography without contrast showing multiple modules with more confluent opacities within the right middle and lower lobes.

The patient was notified immediately of the critical lab values and was referred to the Emergency Department (ED) for further care. He had pancytopenia with mild neutropenia, hyponatremia, and hyperkalemia, renal injury of undetermined chronicity, hypercalcemia, a protein gap, elevated liver-associated enzymes (LAEs), and an elevated lactate dehydrogenase (LDH). In the ED, a computed tomography (CT) chest, abdomen, and pelvis without contrast was performed and showed multiple nodules throughout the lungs with more confluent opacities within the right middle and lower lobes as well as splenomegaly (Figure 1). The patient was admitted to our institution for further workup. The initial concern and working diagnosis by the admitting team was a plasma cell dyscrasia (i.e., multiple myeloma) or a hematologic malignancy and hematology-oncology was initially consulted. Other etiologies that were considered were leukemia, myelofibrosis, systemic viral diagnosis (i.e., Epstein-Barr virus (EBV)), atypical mycobacterial infections, invasive fungal infections, and autoimmune disorders such as sarcoidosis. The patient received fluid resuscitation and allopurinol in consideration for tumor lysis syndrome but ultimately was transferred to an outside hospital (OSH) given the constricted resources at our institution (i.e., inability to perform and initiate dialysis if his renal function did not improve). At the OSH, he required two packed red blood cell (pRBC) transfusions for anemia, and his hypercalcemia was treated with continued fluids, calcitonin, and diuretics. A bone marrow aspirate and core biopsy were obtained and revealed unremarkable flow cytometry and immunophenotyping and hypocellular marrow (40%) with maturing trilineage hematopoiesis, no increase in blasts, and polytypic plasma. In the context of normal serum and urine protein electrophoresis and normal serum free light chains, alternative diagnoses other than plasma cell dyscrasias were then considered. Positron emission tomography-computed tomography (PET/CT) exhibited widespread fluorodeoxyglucose (FDG) avid non-enlarged lymph nodes with splenomegaly and significant splenic hypermetabolic activity as well as innumerable bilateral punctate pulmonary nodules with areas of consolidation demonstrating hypermetabolic activity. Laboratory results from the OSH on hospital day two are shown in Table 2.

**Table 2:** Inpatient Laboratory Workup and Results cells/mm^3:^ cells per cubic millimeters; g/dL: grams per deciliter; cells/µL: cells per microliter; mmol/L: millimoles per liter; mg/dL: milligrams per deciliter; U/L: units per liter; IU/mL: international units per milliliter; mcg/L: micrograms per liter; pg/mL: picograms per milliliter; mg/L: milligrams per liter; mg/24H: milligram per 24 hours

Parameter	Observed Value	Normal Range
WBC	2300 cells	3200-10,800 cells/mm^3^
Hemoglobin	8.3 g/dL	13.1-18.6 g/dL
Platelets	96,000 cells/µL	150,000-350,000 cells/µL
Serum Sodium	134 mmol/L	136-145 mmol/L
Serum Potassium	4.9 mmol/L	3.5-5.1 mmol/L
Serum Chloride	107 mmol/L	98-107 mmol/L
Serum HCO_3_^-^	21 mmol/L	21-32 mmol/L
Serum Urea	22 mg/dL	7-18 mg/dL
Serum Creatinine	2.7 mg/dL	0.7-1.3 mg/dL
Random Blood Glucose	82 mg/dL	70-100 mg/dL
Serum Calcium	10.4 mg/dL	8.5-10.1 mg/dL
Vitamin B12	382 pg/mL	193-986 pg/mL
Serum Magnesium	1.9 mg/dL	1.6-2.6 mg/dL
Serum Total Bilirubin	0.7 mg/dL	0.2-1.0 mg/dL
Serum Alkaline Phosphatase	101 U/L	45-117 U/L
Serum Alanine Aminotransferase (ALT)	16 U/L	16-61 U/L
Serum Aspartate Aminotransferase (AST)	23 U/L	15-37 U/L
HIV 1/2 Ab	Negative	
HIV-1 Antigen (p24)	Negative	
ANA Antibody Screen	Positive	
ANA Ab Pattern	SPECKLED	
ANA Ab Titer	1:160	
Rheumatoid Factor (RF)	24 IU/mL	<10 IU/mL
Angiotensin-Converting Enzyme (ACE)	225 mcg/L	<40 mcg/L
Parathyroid Hormone (PTH)	11 pg/mL	10-65 pg/mL
1,25-dihydroxyvitamin D	70 pg/mL	18-78 pg/mL
C-Reactive Protein	18.5 mg/L	<3.0 mg/L
24-hour Urine Protein Collection (total)	4210 mg/24H	<150 mg/24H

In the context of his elevated ACE level, hypercalcemia, and renal injury with nephrotic-range proteinuria, there was a suspicion of an infiltrative process such as sarcoidosis. He then underwent a CT-guided lung core biopsy with pathology revealing non-caseating granulomatous inflammation with numerous multinucleated giant cells in a background of fibrosis consistent with sarcoidosis. Stains were negative for atypical mycobacteria and fungal infections. These results were supportive of a diagnosis of presumed sarcoidosis with lung involvement and apparent renal sarcoidosis that could explain the patient’s symptoms and laboratory findings. Given the patient’s nephrotic-range proteinuria, he was placed on an angiotensin II receptor blocker (ARB).

Upon discharge, his calcium levels had improved, and serum creatinine improved to 2.6 mg/dL although the patient was still pancytopenic. The patient’s discharge labs are shown in Table 3. He felt well with no complaints. He was discharged on 60 mg of prednisone to be taken daily and was prescribed amlodipine and losartan for hypertension and nephrotic-range proteinuria management. In addition, due to anticipated long-term corticosteroid use, he was prescribed *Pneumocystis jirovecii* pneumonia (PJP) prophylaxis with atovaquone and a proton pump inhibitor. He had follow-up appointments with Pulmonology, Rheumatology, and Nephrology. In his follow-up with Rheumatology, the patient was eventually placed on adalimumab subcutaneously due to intolerable side effects from corticosteroids (weight gain, malaise) as well as lack of improvement with corticosteroids. Ultimately, the patient’s diagnoses and the requirement for long-term injectable medications classified him as indefinitely non-worldwide deployable, and he was ultimately medically separated from the military.

**Table 3:** Day of Discharge Laboratory Parameters cells/mm^3^: cells per cubic millimeters; g/dL: grams per deciliter; cells/µL: cells per microliter; mmol/L: millimoles per liter; mg/dL: milligrams per deciliter; U/L: units per liter

Parameter	Observed Value	Normal Range
WBC	2700 cells	3200-10,800 cells/mm^3^
Segmented Neutrophils	65.7%	44.5-78.4%
Hemoglobin	8.7 g/dL	13.1-18.6 g/dL
Platelets	112,000 cells/µL	150,000-350,000 cells/µL
Serum Sodium	135 mmol/L	136-145 mmol/L
Serum Potassium	3.8 mmol/L	3.5-5.1 mmol/L
Serum Chloride	105 mmol/L	98-107 mmol/L
Serum HCO_3_^-^	24 mmol/L	21-32 mmol/L
Serum Urea	22 mg/dL	7-18 mg/dL
Serum Creatinine	2.6 mg/dL	0.7-1.3 mg/dL
Random Blood Glucose	90 mg/dL	70-100 mg/dL
Serum Calcium	9.9 mg/dL	8.5-10.1 mg/dL
Corrected Calcium	10.5 mg/dL	
Serum Total Protein	8.3 g/dL	6.4-8.2 g/dL
Serum Albumin	3.3 g/dL	3.4-5.0 g/dL
Serum Phosphorus	3.5 mg/dL	2.5-4.9 mg/dL
Serum Magnesium	2.0 mg/dL	1.6-2.6 mg/dL
Serum Total Bilirubin	0.8 mg/dL	0.2-1.0 mg/dL
Serum Alkaline Phosphatase	119 U/L	45-117 U/L
Serum Alanine Aminotransferase (ALT)	19 U/L	16-61 U/L
Serum Aspartate Aminotransferase (AST)	25 U/L	15-37 U/L

## Discussion

Sarcoidosis is a multisystem inflammatory disease that causes noncaseating granulomatous infiltration of tissues, and almost any organ can be affected [5]. The vast majority of individuals with sarcoidosis have lung involvement, but 30-50% of patients have extrapulmonary involvement and this is more likely to occur in African Americans as in our patient [4]. The peak age of onset is between 20 and 39 years of age [3]. Extrapulmonary sarcoidosis may have a significant overlap of clinical presentations with other disease processes making the diagnosis and recognition difficult [6]. The literature reports that 1%-5% of individuals with sarcoidosis have renal involvement, although the true prevalence is not known and may be higher as the disease course progresses [5]. In this way, due to the clinical rarity, renal and urologic involvement may be an overlooked component when making the diagnosis of sarcoidosis. Furthermore, due to the phenotypic heterogeneity, sarcoidosis is a diagnostic challenge with an overlap of clinical presentations of other disease processes. The patient described in this case illustrates rare extrapulmonary manifestations of sarcoidosis, highlights the importance of avoiding premature closure of the differential diagnosis, and provides insight into military medicine decision-making.

The case described herein is a rarer initial manifestation of sarcoidosis as the patient’s extrapulmonary manifestations were primarily renal and hematologic, rather than the more common manifestations of skin lesions, lymphadenopathy, arthritis, and uveitis. Hematologic abnormalities in sarcoidosis may be secondary to hypersplenism, bone marrow suppression, or destruction via autoimmune mechanisms [7]. Renal sarcoidosis can present in a variety of ways including hypercalcemia and hypercalciuria, nephrolithiasis and nephrocalcinosis, granulomatous interstitial nephritis, and glomerular disease and tubular dysfunction [3]. Our patient had hypercalcemia and nephrotic-range proteinuria with renal injury, the latter indicating a nephropathy which is an even more infrequent presentation of renal sarcoidosis. Although our patient did not receive a kidney biopsy to distinguish between histologic diagnoses, membranous nephropathy, focal segmental sclerosis, and glomerulonephritis have been described as possible manifestations in the literature [3]. However, generally, this does not change management as glucocorticoids are the mainstay of therapy even in extrapulmonary sarcoidosis and particularly in renal sarcoidosis with hypercalcemia as it helps to normalize calcium levels [3].

Recognizing and diagnosing sarcoidosis is difficult in the context of its wide range of clinical manifestations and ability to affect many organ systems. However, early recognition and prompt diagnosis allow for earlier treatment which may help prevent irreversible organ dysfunction and life-threatening complications. In our case, the clinical suspicion of a plasma cell dyscrasia or hematologic malignancy and the overlap of presentations led to an asynchronous evaluation, premature closure of the differential diagnosis, and ultimately a possible delay of diagnosis and treatment initiation for several days. For example, the patient may have received a CT-guided lung biopsy days earlier rather than waiting for the bone marrow biopsy to result. When a patient presents with an acute illness necessitating inpatient care and causing organ damage (in our case renal, electrolyte, and hematologic dysfunction), it’s important to combat the clinical inertia of anchoring bias and to ensure the execution of clinical reasoning and a synchronous evaluation to prioritize differential diagnoses and to avoid diagnostic inaccuracies and delay of treatment. One could postulate that in the case described, a tissue or lung biopsy may have been considered earlier in the hospitalization course prompting glucocorticoid initiation, improvement of symptoms and organ dysfunction, and an earlier hospital discharge.

Specific to military medicine, systemic diseases such as sarcoidosis and their respective treatments in an active-duty service member may have implications on the service member’s ability to execute the worldwide deployability required of a military member. For example, active-duty service members can be deployed to very remote, austere environments worldwide which cannot support the proper storage or administration of certain medications or may increase the risk of complications and death given the lack of easily accessible medical capabilities. This is critical to know as a civilian physician, as many of our service members receive their care in the civilian sector, as it may change education and counseling performed to the patient on occupational expectations and could change treatment options. Frequently this comes into play for individuals who require intravenously administered or injectable medications, as austere environments cannot reliably administer or store these medications. For example, our patient required adalimumab which was not compatible with the ability to deploy on a moment’s notice. As such, certain disease processes and required treatments thereafter may require medical separation from the military of a service member to maintain a worldwide deployable force and to avoid an increased risk to service members based on occupational requirements.

## Conclusions

In conclusion, this case report highlights unique and rare manifestations of sarcoidosis and the importance of avoiding anchoring bias and prematurely closing the differential diagnosis to avoid diagnostic error and to expedite the correct management in a patient. Sarcoidosis presentations may overlap with many other disease processes and should be kept on the differential until definitively ruled out. Additionally, this case provides insight into the clinical decision-making in military medicine in evaluating a patient for continued service in the armed forces in the context of their specific disease process and treatments required to maintain a medically ready and worldwide deployable force.
